# Biliary Mucinous Cystic Neoplasm of the Liver with Ovarian Stroma and Elevated Serum and Cystic Fluid Cancer Antigen 19-9 Levels

**DOI:** 10.7759/cureus.1863

**Published:** 2017-11-20

**Authors:** Georgios P Fragulidis, Eirini V Pantiora, Elissaios A Kontis, Elias Primetis, Andreas Polydorou, Eleni Karvouni, George Polymeneas

**Affiliations:** 1 2nd Department of Surgery, ARETAIEIO Hospital, National and Kapodistrian University of Athens School of Medicine; 2 1st Department of Radiology, ARETAIEIO Hospital, National and Kapodistrian University of Athens School of Medicine; 3 Department of Pathology, ARETAIEIO Hospital, National and Kapodistrian University of Athens School of Medicine

**Keywords:** ovarian stroma, liver cyst, biliary mucinous cystic neoplasm, liver resection, ca19-9

## Abstract

Biliary mucinous cystic neoplasms of the liver are rare cystic tumors comprising less than 5% of the liver cystic neoplasms. These tumors demonstrate a female predominance and entail a risk of malignant transformation. We present a 56-year-old female patient with a multiloculated liver cystic lesion measuring 22 cm who underwent a cystectomy with en bloc resection of the liver segments II, III, and cholecystectomy. Serum cancer antigen 19.9 was 4,122.00 U/ml, supporting the diagnosis of a biliary cystic tumor. The cytology of the cystic fluid was negative for malignancy and intracystic fluid cancer antigen 19.9 level was measured over 12,000.00 U/l. The patient is free of recurrence at one-year follow up. Although a rare entity, the biliary mucinous cystic neoplasms should be considered in the differential diagnosis in the patients with liver cystic tumors. The appropriate management with complete surgical resection with negative margins is recommended given the risk of recurrence and malignant transformation.

## Introduction

Biliary mucinous cystic neoplasms (BMCNs) are slow growing rare cystic lesions of the liver. They are mostly presented in the fifth decade of life and occur more commonly in females, in approximately 90% [1]. The clinical presentation varies and is usually atypical and differential diagnosis from other cystic lesions remains challenging despite progress achieved in the radiological modalities. The proper management requires complete resection, considering they have a tendency of recurrence and malignant transformation as 20% may become invasive [2]. We report the management of a female patient with a giant biliary mucinous cystic neoplasm of the liver and elevated serum cancer antigen (CA19-9).

## Case presentation

**Figure 1:**
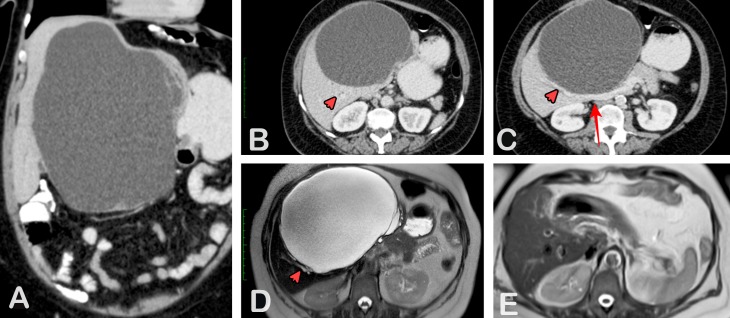
The preoperative contrast enhanced the by the computed tomography and magnetic resonance imaging. a: coronal reconstruction, b and c: axial slices and d: the T2W magnetic resonance imaging, demonstrates a large cystic mass with the maximum diameter of 21.5 cm. There are a few thin internal septations that show mild contrast enhancement but no focal wall thickening or other solid components in the mass. The lesion occupies II, III, IV, V ,and VIII hepatic segments and displaces intrahepatic vessels with no sign of vascular invasion (red arrow: main portal vein). The intrahepatic ducts at the periphery of the mass are slightly dilated due to the mass effect (red arrowheads), e: the T2W magnetic resonance imaging after the lesion excision.

A 56-year-old female patient was referred to our hospital for a large cystic hepatic tumor. The tumor was incidentally diagnosed eight years ago by the abdominal ultrasound consistent as a simple cyst and no intervention was recommended at that time. The patient has remained under clinical observation ever since. Currently, she developed an abdominal discomfort and she noticed that her abdominal girdle appeared to be slowly increasing in size. The physical examination showed an indurated mass in the right upper quadrant extending approximately 15 cm below the right costal margin. Complete blood count and comprehensive metabolic panel were normal. Serum cancer antigen 19.9 (CA 19.9) was 4,122.00 U/ml, (n.v. < 35 U/ml), supporting the diagnosis of a cystic tumor. The abdominal imaging (the computed tomography (CT) scan and magnetic resonance imaging (MRI)) revealed a multiloculated cystic lesion of the liver with internal septae measuring 22 cm. The cyst was located in the center of the liver with perihilar mass effect and profound distortion of the left pedicle as can be seen in Figure 1.

**Figure 2:**
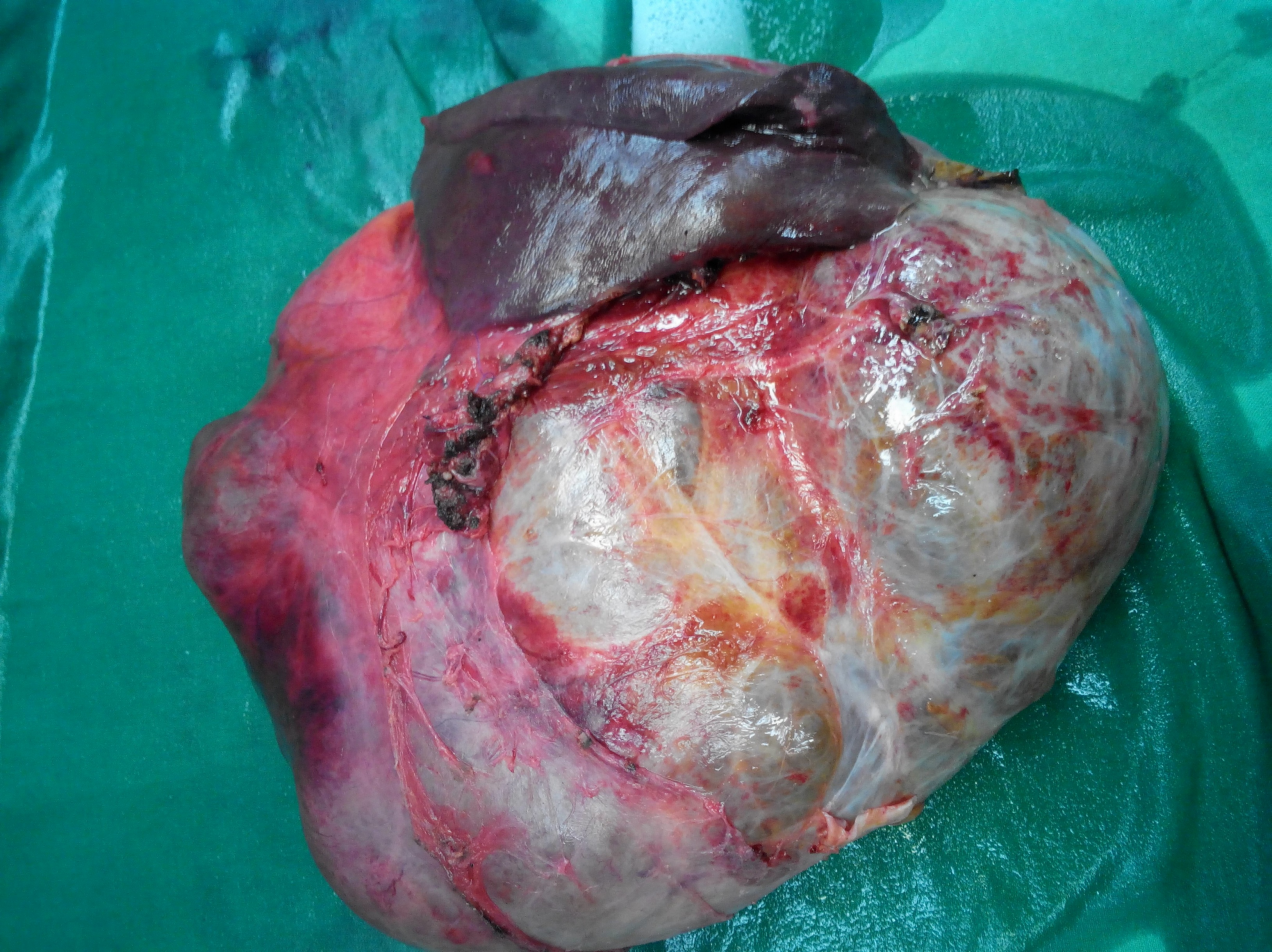
The external view of the specimen including the cystic tumor and the resected segments II, III of the liver.

Intraoperative, the cystic tumor occupied almost completely the left lateral lobe and was extended centrally over the hepatic hilum thrusting the porta hepatis, the gallbladder and the liver segments II and III. The right liver hilar pedicles were carefully dissected and the patient underwent cystectomy and en bloc resection of the liver segments II, III and cholecystectomy (Figure 2).

**Figure 3:**
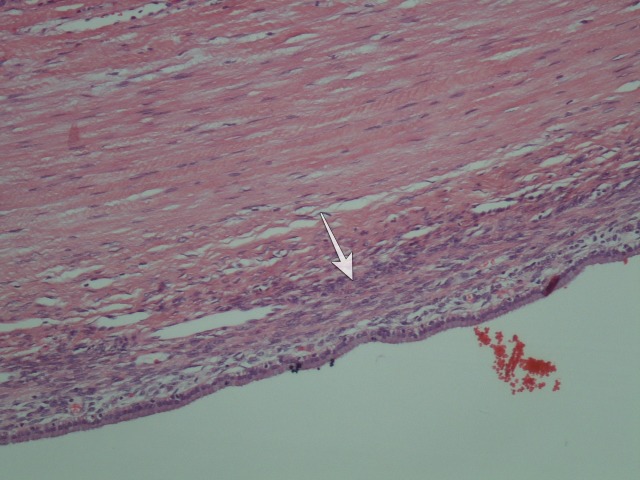
The hematoxylin-eosin (x 100) stained histologic section. The ovarian-like stroma is noted (white arrow).

Postoperative, a grade B bile leakage was successfully managed with the endoscopic sphincterotomy and she was discharged home on postoperative day seven. The cytology of the cystic fluid was negative for malignancy and the intracystic CA 19.9 level was measured over 12,000.00 U/l, (n.v. < 35 U/ml). The pathology report of the specimen revealed a low-grade BMCN with a typical ovarian stroma (Figure 3).

**Figure 4:**
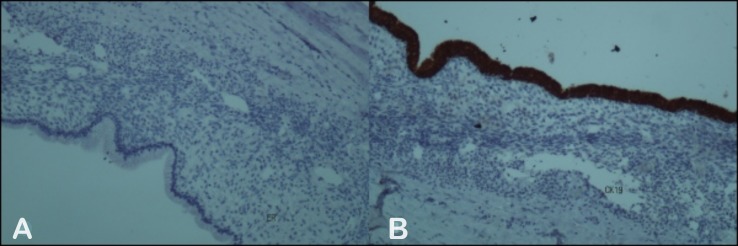
The immunohistochemistry, A: to estrogen receptor (ER) and B: to cytokeratin 19 (CK19).

Immunostaining was negative to estrogen receptor (ER) (Figure 4a) and positive to cytokeratin 19 (CK19) (Figure 4b).

The patient was free of recurrence at one-year follow up which was confirmed by the clinical examination and MRI imaging.

## Discussion

Biliary mucinous cystic neoplasms (BMCNs) are rare tumors occupying a small percentage of less than 5% of all the cystic lesions of the liver. These lesions have been reported under the terms of ‘biliary cystadenoma’ and ‘biliary cystadenocarcinoma’. In 2010, the World Health Organization (WHO) had categorized the biliary cystadenoma into BMCNs with low-, intermediate-, or high-grade intraepithelial neoplasia and the biliary cystadenocarcinoma into BMCNs with an associated invasive carcinoma [1]. The presence of an ovarian stroma was accepted as a requirement for the diagnosis of BMCNs. However, most authors continue using the terms cystadenoma and cystadenocarcinoma to describe this entity.

Due to the rarity of this entity, their true prevalence, and the origin remains unclarified. The two principal theories are that they derive from ectopic intrahepatic ovarian tissue or from ectopic intrahepatic embryonic gallbladder rests because of the presence of epithelial cells resembling primitive hepatobiliary cells [3]. The BMCNs have a creatine kinase (CK) profile that supports an origin from the biliary tract, while the finding of the ER positivity in the ovarian stroma supports its hormonal dependence and explains its exclusive occurrence in the females [2]. The presence of the ovarian stromal component may impart a hormone-mediated tumor suppressor effect on the transformation to malignancy. The lack of the ovarian stroma is associated with decreased median survival compared with tumors expressing stroma. However, even in studies restricted to BMCNs with ovarian stroma, 17–19% of the tumors were found to be malignant [4]. A malignant transformation rate of up to 20% has been reported and the invasive BMCNs accounts for 0.41% of the malignant hepatic epithelial tumors [1].

The clinical presentation varies since many patients are asymptomatic or present with non-specific symptoms. The serum tumor markers such as CA-19.9 and carcinoembryonic antigen (CEA) seem to be of no particular help since they cannot differentiate invasive BMCNs or even simple cysts [1]. The highly elevated levels of serum tumor markers which were measured in our case confirm the inadequacy of these biochemical parameters to contribute to the differential diagnosis of the cystic hepatic lesions in terms of malignancy.

Regarding the differential diagnosis of the liver cyst, liver abscess and hydatid cyst are the two entities reported as the most likely to be confused with BMCNs. Preoperative imaging studies are currently the most reliable methods of diagnosing BMCNs and differentiates it from other cystic lesions, such as simple cysts, hydatid cyst, liver abscess or biliary intraductal papillary mucinous neoplasm. Nevertheless, it has been reported that correct preoperative radiological diagnoses are made in < 50% of the cases [1]. Imaging studies usually reveal a solitary complex cystic mass with internal septa, mural or septal nodules and papillary projections, which may enhance after intravenous contrast administration. The BMCNs exhibit a stronger mass effect on imaging because of their viscous fluid compared with simple hepatic cysts containing serous fluid. Histologically, it has been a controversy regarding the distinction between BMCNs and a unique biliary tumor named intraductal papillary mucinous neoplasm of the bile duct with biliary tree communication and no ovarian stroma. Since the presence of ovarian stroma was accepted as a prerequisite for the diagnosis of BMCNs, the distinct entity of biliary intraductal papillary mucinous neoplasms was proposed [4]. However, more than a few reports of cystic liver tumors have been published where both ovarian stroma and bile duct communication are not detected [5].

The standard treatment of the biliary BMCNs is complete resection. Simple interventions such as the percutaneous aspiration, sclerotherapy, surgical fenestration have a high recurrence rate and a delay in diagnosis, particularly when no histology is obtained. The frozen section examination of enucleated tumors must be performed because resection of the adjacent parenchyma may follow in the cases with positive results. Complete resection with negative margins is the appropriate management with reasonable long-term results. The patients with invasive BMCNs have a better prognosis than those with hepatocellular carcinoma or cholangiocarcinoma, which emphasizes the importance of distinguishing between disease types [1].

## Conclusions

Biliary mucinous cystic neoplasms (BMCN) should be considered in the differential diagnosis in the patients with liver cystic tumors. Because of the high recurrence rate and difficult accurate preoperative diagnosis, the formal liver resection is mandatory to remove the occult malignancy. The enucleation with free margins is an option and is indicated where resection is technically not feasible.
